# Does the Stockholm Syndrome affect female sex workers? The case for a “Sonagachi Syndrome”

**DOI:** 10.1186/s12914-018-0148-4

**Published:** 2018-02-06

**Authors:** Abraar Karan, Nathan Hansen

**Affiliations:** 1 Brigham and Women’s Hospital, Harvard Medical School, Boston, Massachusetts USA; 2 Department of Health Promotion and Behavior, University of Georgia College of Public Health, Athens, Georgia USA

## Abstract

Female sex workers are subjected to intense physical, sexual, and mental abuses that are well documented in the medical and public health literature. However, less well-studied are the mental coping mechanisms that are employed by women in this population to survive. The Stockholm Syndrome has been discussed in the news media as a potential phenomenon in this vulnerable population, but has not been formally studied. From a previous retrospective qualitative analysis reviewing interviews with women in sex work throughout India, we found that the four main criteria for Stockholm Syndrome (perceived threat to survival; showing of kindness from a captor; isolation from other perspectives; perceived inability to escape) are present in narrative accounts from this population. Thus, we propose that Stockholm Syndrome should be considered as a contributing phenomenon with regard to the psychological challenges faced by female sex workers, and can likely help guide interventions accordingly.

## Background

An unusual phenomenon has been observed in female sex workers (FSW) around the world, many of whom have been kidnapped, trafficked, emotionally abused, sexually violated, physically exploited, and isolated from their families and the public. When rescued by law enforcement and supported by non-governmental organizations, they refuse to testify in court against their traffickers. This has been reported in both the United States and England, and was a prevalent concern with leading anti-trafficking NGO staff in India, who author AK had interviewed [1, 2]. Currently, the closest psychiatric descriptors of trauma endured by female sex workers are Complex Post Traumatic Stress Disorder, or Disorders of Extreme Stress Not Otherwise Specified (DESNOS), but neither of these have been included in the DSM IV due to debate over whether they are distinctly different from Post Traumatic Stress Disorder as it currently is defined [3–5]. We propose that a further explanation of this behavior is the Stockholm Syndrome, a phenomenon in which those held captive develop bonds with their captors in what is thought to be a survival and coping strategy [6, 7]. Of note, the condition also has not been accepted as a true medical syndrome by the medical or psychiatric community, in part because of its rarity and the very specific vulnerable groups among which it has been documented. The Stockholm Syndrome has emerged as a pattern of behavior exhibited by those in situations of captivity, including hostage situations (bank robberies; plane hijackings), abusive domestic relationships, and sexual abuse in children [8, 9]. The specific conditions to which female sex workers are subjected have not been studied in relation to the Stockholm Syndrome. In this debate piece, we propose that the Stockholm Syndrome as it is currently understood may be a real contributor to the psychological challenges that plague female sex workers, particularly with regard to attempts at rehabilitation and ending dependency from captors.

## Main text

In our research with Indian female sex workers (FSWs), we retrospectively studied narrative accounts of interviews with women in this population who were part of empowerment groups organized by the nonprofit Apne Aap Women Worldwide (AAWW). AAWW operates in several states in Northern India with a variety of communities in brothel, street, and home-based sex work. We analyzed interviews conducted between June 2010 and April 2012 by AAWW with female sex workers in Delhi, Bihar, Kolkata, Mumbai and Rajasthan, India. The interviews were conducted by staff members of Apne Aap as part of a monthly newsletter known as the *Red Light Dispatch.* Apne Aap received informed consent from all women as part of their organizational protocol to publish these interviews in their monthly newsletter, which is distributed widely to donors, volunteers, and other supporters of the organization. We used NVivo coding software and a grounded theory approach to highlight themes related to mental health and trauma. The full methods and analysis of these interviews are reported in a separate research paper [8]. In our qualitative analysis, we were struck by patterns of abuse that were consistent with the pre-conditions for Stockholm Syndrome that have been described by Graham et al. [9] (Table 1) The components of initiation and continuation of sex work involved physical and sexual violence, physical isolation, psychological demoralization; and perhaps most notably, the presence of a love relationship with the trafficker.

**Table 1:** Pre-conditions for Stockholm Syndrome in female sex workers

Pre-Condition of Stockholm Syndrome (Graham et al.)	Conditional Similarities in Female Sex Worker Environments	Analysis of Pre-Conditions of Stockholm Syndrome in Female Sex Workers
Perceived Threat to Survival and Belief that Captor is Willing to Carry-Out the Threat	*“I can leave but I need the police to protect me because there are many people who can torture me. I don’t know who they are but they come in the night and they rape us. Once I was kidnapped, stabbed, and raped just because I refused to open my door for prostitution at midnight.”*	Female sex workers are subjected to extreme violence after they are trafficked, especially in the initial months upon arrival to the brothel. This violence comes from both clients, as well as those who work in the brothel.
Victim’s Perception of Kindness from Captor within Context of Terror	*“He was very nice to me during the initial days but then he asked me to present myself before a man to whom I was sold…I realized that the person I was so much in love with and had trusted with all my heart was acting as a pimp and presenting me to people and earning lots of money out of it.”*	Many trafficking victims are initially lured by men through duplicitous romantic relationships. For many, this hope of love and kindness remains a factor as they continue interacting with the trafficker even while working in the brothel.
Isolation from Perspectives Other than that of the Captor	*“Girls and women were kept in constant fear. They were not allowed to talk to each other and were socially brain washed.”* *“One of the realities of life in a Red Light Area is seclusion. You are brought into the Red Light Area (RLA) as a child and for years kept locked inside a room with only the Malkin to talk to. It’s only after about ten years or more that you can start going out and talking to the other women.”*	Most trafficked women shared narratives of complete physical isolation from other girls and the public (aside from clients). This tactic is used to prevent girls from escaping or seeking help. It contributes to the demoralization of the victim.
Perceived Inability to Escape	*“There were many girls who tried to rise against the brothel owner and escape. But they would be found and treated even worse than before. I saw the fate of rebels in brothels and their hardships killed my courage to run away.”*	After physical violence and isolation for several months, many women shared that they did not believe escape was possible. They also witnessed examples of others who tried and failed, which further made the idea of escaping an unlikely if not impossible option.

The first precondition of Stockholm Syndrome has been described as a perceived threat to survival and the belief that a captor would carry such threat to completion. For many women who are trafficked, physical violence and torture are central aspects of their experience. (Table 1) The threat to survival is perpetrated by both brothel workers (pimps, traffickers, brothel madams), as well as clients. In many narratives, women discussed that death was a plausible result of starvation, physical violence, and sexual violence. The second precondition is the showing of love or kindness to the victim by the captor. This is particularly notable in that many women who are trafficked continue to have a relationship with their trafficker, or others develop relationships with clients. (Table 1) Some women, for instance, described having hope that they would be able to start a family with their trafficker or a client. However, within the context of Stockholm Syndrome, this criterion essentially refers to any action which might help the woman to survive. As the FSW’s survival is essential to the running of the sex market, pimps and brothel owners naturally must provide the woman with enough food and shelter for her to be healthy enough to work. Thus, the nature of the pimp-prostitute relationship is in some ways inherently designed to allow for kindness or demonstrations of positive interactions. Moreover, abuses from pimps and brothel madams are lessened as the woman accepts her position in the brothel and it is likely that her treatment improves over time. A third precondition is that victims are isolated from the outside world. Many women described that their first several months in the brothel were completely isolated aside from seeing a brothel madam or trafficker. (Table 1) There was no contact with the outside world, creating a sense of depersonalization and demoralization. The final precondition for Stockholm Syndrome is the perceived inability to escape. Female sex workers who tried to escape from the brothel were physically beaten in front of other women to dissuade them from trying to escape (Table 1).

For many trafficked women, entry into sex work was through false promises of marriage or of a well-paying job in a different city. Once in sex work, many women felt that the stigma of their circumstance would prevent their families from ever taking them back, or allowing them to have a normal role in public society. Furthermore, corruption and sexual abuse from law enforcement officials (who are often also clients) further convinced women that there was no outside help available. We believe that these extreme circumstances compel women to focus on survival rather than escape, which is essentially the crux of the Stockholm Syndrome—a psychological attempt to physically survive in captivity.

This pattern of behavior is not only restricted to brothel-based prostitution as similar relationships are also present in street, home, and caste-based prostitution scenarios. This has been noted as a point of difficulty for anti-trafficking rehabilitation programs around the world [1].

## Conclusions

The understanding of female sex workers’ psychological state as it relates to the Stockholm Syndrome must be further studied in this population. Understanding the proposed psychology of Stockholm Syndrome in this population is essential because it will inform rehabilitation efforts and windows for intervention that can disrupt the emotional dependencies that are being described. For instance, psychotherapy specific to traumatic psycho-dependence may be an integral part of FSW rehabilitation, and the presence of Stockholm Syndrome preconditions should be considered in each individual victim’s case. Furthermore, if the Stockholm Syndrome is indeed significant, it would have substantial implications for legal cases in which rescued women refuse to testify against captors. For instance, requiring women to participate in legal proceedings that could be influenced by positive feelings toward captors that are rooted in trauma would need to considered with special exception. We propose a differentiation of the Stockholm Syndrome in female sex workers, more specifically to be referred to as the “Sonagachi Syndrome” (Sonagachi is the largest Red Light District in India, located in the city of Kolkata).
